# The PSMP-CCR2 interactions trigger monocyte/macrophage-dependent colitis

**DOI:** 10.1038/s41598-017-05255-7

**Published:** 2017-07-11

**Authors:** Xiaolei Pei, Danfeng Zheng, Shaoping She, Jing Ma, Changyuan Guo, Xiaoning Mo, Yingmei Zhang, Quansheng Song, Yu Zhang, Dalong Ma, Ying Wang

**Affiliations:** 1 Department of Immunology, School of Basic Medical Sciences, and Key Laboratory of Medical Immunology of Ministry of Health, Peking University Health Science Center, Beijing, 100191 P.R. China; 2 Center for Human Disease Genomics, PekingUniversity, Beijing, 100191 P.R. China; 3 Institute of Hematology and Blood Diseases Hospital, Chinese Academy of Medical Sciences and Peking Union Medical College, 288 Nanjing Road, Tianjin, 300020 P.R. China; 4 Department of Pathology, Cancer Institute and Hospital, Chinese Academy of Medical Sciences, 17 Panjiayuan South Lane, Chaoyang District, Beijing, 100021 P.R. China

## Abstract

Monocytes/macrophages have been found to be an important component of colitis. However, the key chemokine that initiates the CCR2^+^ monocytes migration from circulation to colitis tissue remains to be undiscovered. PC3-secreted microprotein (PSMP) is a novel chemokine whose receptor is CCR2. The physiological and pathological functions of PSMP have not yet been reported. In this study, PSMP was found to be expressed in colitis and colonic tumor tissues from patients and significantly up-regulated in mouse DSS-induced colitis tissues. PSMP overexpression in the colon aggravated the DSS-induced colitis and the anti-PSMP neutralizing antibody mollified the colitis by reducing macrophage infiltration and inhibiting the expression of IL-6, TNF-α and CCL2. Furthermore, we demonstrated that lipopolysaccharide and muramyl dipeptide induced PSMP expression in the colonic epithelial cells. PSMP was up-regulated in the initial stage prior to IL-6, TNF-α and CCL2 up-regulated expression in DSS colitis and promoted the M1 macrophages to produce CCL2. PSMP chemo-attracted Ly6C^hi^ monocytes in a CCR2 dependent manner via *in situ* chemotaxis and adoptive transfer assays. Our data identify PSMP as a key molecule in ulcerative colitis, which provides a novel mechanism of monocyte/macrophage migration that affects gut innate immunity and makes PSMP a potential target for controlling colitis.

## Introduction

Inflammatory bowel disease (IBD), including ulcerative colitis (UC) and Crohn’s disease, is recognized inflammatory disorders in the colonic tract^1^. IBD-related carcinogenesis is one of the most common cancers around the world^2^. Patients with IBD have been reported to exhibit a high risk of acquiring colonic cancer^3, 4^. However, the mechanisms of IBD are still not fully understood. There are many reports from different research teams that have revealed that monocytes, monocyte-derived macrophages and dendritic cells (DCs) all play key roles in maintaining colonic homeostasis and regulating colonic inflammation. Immune cells, as well as colonic epithelial cells and smooth muscle cells, have been reported to contribute to the entire process of colonic inflammation^5–8^.

Monocytes in circulation are a group of immune cells that are chemo-attracted and migrate into inflammatory tissue in the early stage of inflammation^9^. Once the newly extravagated monocytes are stimulated by microbacteria and the cytokines from resident immune cells or epithelial cells, they transform into inflammatory macrophages (i.e., classical macrophages or M1 macrophages) to promote colonic inflammation via the production of the pro-inflammatory cytokine IL-6, CCL2 and TNF-α. Furthermore, these early-arriving monocytes have been found to be Ly6C^hi^CCR2^+^CX3CR1^mid^, but the key chemokine that initiates the migration of Ly6C^hi^CCR2^+^CX3CR1^mid^ monocytes from circulation into the tissue has been unknown yet^10–13^.

CCR2 is defined as one of the classical chemokine receptors, which belong to the type A G protein-coupled receptor superfamily. CCR2 has been reported to be important and essential for inflammatory monocyte promotion during inflammation. CCR2 performs a key role in the chemotactic procession in immune cells, which further influence immune homeostasis and inflammation. CCR2 has been reported to be an essential chemokine receptor that aids the entry of bone-marrow residing monocytes into the bloodstream and the migration of myeloid-derived cells into the lamina propria^14–17^.

CCL2 is a well-known ligand of CCR2. Regarding CCL2, the primary sources of its expression had been reported to be monocytes and “classical pro-inflammatory” macrophages^18–20^. One team found that the induction of colitis in CCL2 knockout (KO) mice was similarly to wild type mice^21^, however, the CCR2 KO mice exhibited mollified colitis, which indicated that the effect of CCR2 on colitis was independent on CCL2^21^.

PSMP, namely PC3-secreted microprotein, was initially found in PC3 cells and benign and malignant prostate tissues. Our previous study using omics strategies reveals PSMP a novel chemokine^22^. PSMP exhibits chemotaxis for peripheral blood monocytes and lymphocytes in a CCR2-dependent manner *in vitro*. The affinity between PSMP and CCR2 was found to be comparable to that between CCL2 and CCR2^23^. However, the physiological and pathological functions of PSMP have not yet been elucidated. Here, we demonstrated that PSMP was expressed in the colonic mucosa of patients with colitis and significantly up-regulated in the initial stage prior to the expression of IL-6, TNF-α and CCL2 in a DSS-induced colitis in mice. Furthermore, our results revealed that PSMP induced chemotaxis of Ly6C^hi^ monocytes *in vivo*, PSMP promoted DSS-induced colitis and a neutralizing antibody of PSMP mollified colitis by reducing macrophage accumulation.

## Results

### PSMP chemo-attracts Ly6C^hi^ monocytes in a CCR2-dependent manner *in vivo*

Although our previous study demonstrated that PSMP can chemo-attract monocytes in a CCR2-dependent manner *in vitro*, it remained essential to determine whether PSMP’s chemotactic function on monocytes depends on CCR2 *in vivo*.

**Figure 1:**
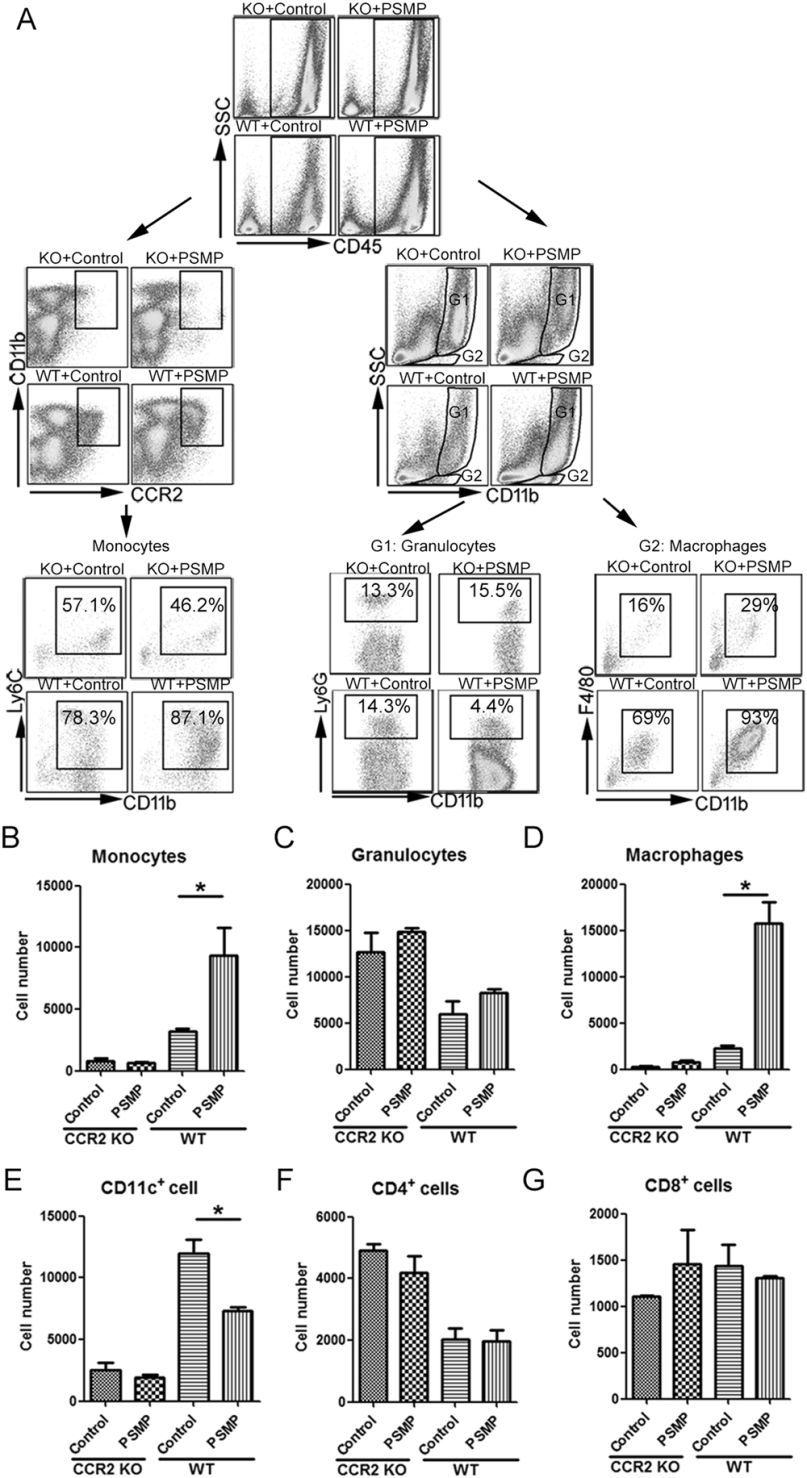
PSMP protein chemo-attracted the CCR2^+^Ly6C^hi^ monocytes into the abdominal cavity. (**A**) Forty-eight hours after the abdominal injection of PSMP protein, the main immune cells that were chemo-attracted by PSMP were analyzed by flow cytometry with the antibodies of CD45, CD11b, CCR2, Ly6C, Ly6G and F4/80. (**B**–**G**) The monocytes (CD11b^+^CCR2^+^Ly6C^hi^), granulocytes (G1&CD11b^+^Ly6G^+^), macrophages (G2&CD11b^+^F4/80^+^), CD11c^+^ cells, CD4^+^ cells and CD8^+^ cells in the abdominal cavities of the wild type and CCR2 KO mice intraperitoneally injected with and without PSMP were analyzed by flow cytometry system. Data were representative of at least two independent experiments with four mice per group. *0.01 < *p* < 0.05, **0.001 < *p* < 0.01, and ***p < 0.001.

Following the abdominal injection of PSMP protein, PSMP chemo-attracted the CD11b^+^Ly6C^hi^CCR2^+^ monocytes (“pro-inflammation”) into the abdominal cavity (Fig. 1A) and significantly more in wild type mice than other groups (Fig. 1A and B). Macrophages and granulocytes were distinguished by CD11b-F4/80 and CD11b-Ly6G markers (Fig. 1A). In wild type mice abdominal cavity, macrophages with PSMP existing were more than the control group, which did not accumulate in CCR2 KO mice no matter with or without PSMP existing (Fig. 1D).

Furthermore, granulocytes, CD11c^+^ cells, CD4^+^ cells and CD8^+^ cells were respectively analyzed. The results showed that there was no significant accumulation of CD11c^+^ cells, granulocytes and T cells with or without PSMP in wild type mice (Fig. 1C,E,F and G). In CCR2 KO mice abdominal cavity, monocytes, macrophages, granulocytes, T cells and CD11c^+^ cells showed no difference no matter with or without PSMP existing (Fig. 1B–G).

**Figure 2:**
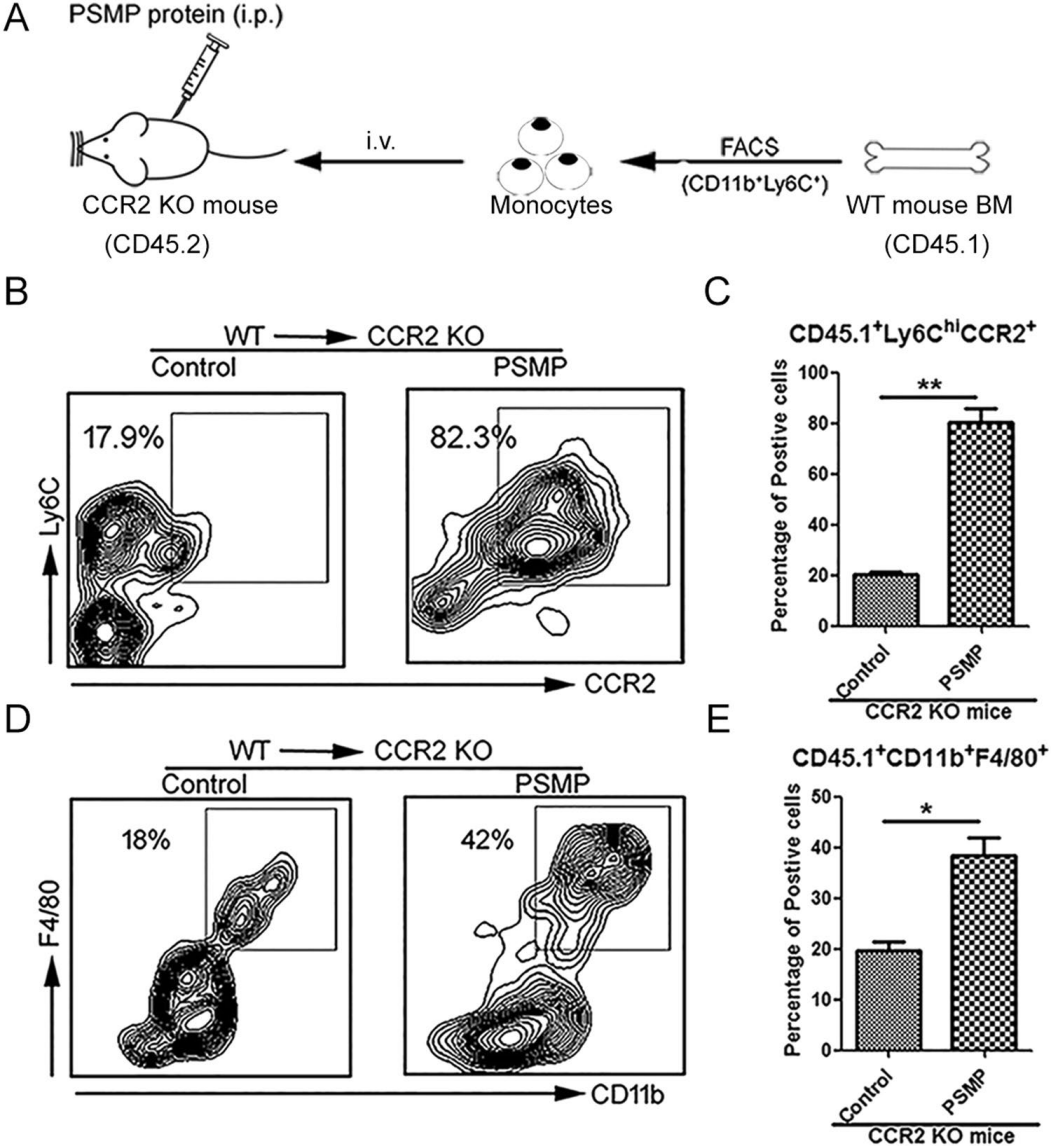
PSMP protein could specifically chemo-attracted the CCR2^+^Ly6C^hi^ monocytes into the abdominal cavity with CCR2 dependent manner. (**A**) Ly6C^hi^ monocytes were sorted from wild type mice’s bone marrow, infused into CCR2 KO mouse (1 × 10^7^ cells per mouse) by *i*.*v*. and PSMP protein was injected into the abdominal cavity at the same time. (**B**) and (**C**) Forty-eight hours after the CCR2^+^Ly6C^hi^ monocytes from wild type mouse bone marrow were intravenously introduced into the circulation of the CCR2 KO mice, and the percentages of monocytes (CCR2^+^Ly6C^hi^) in the abdominal cavities with and without PSMP were detected and statistically analyzed by flow cytometry. (**D**) and (**E**)The percentages of macrophage (CD11b^+^F4/80^+^) in the abdominal cavities of the adopted transfer mice with and without PSMP were analyzed by flow cytometry. Data were representative of at least two independent experiments with four mice per group. *0.01 < *p* < 0.05, **0.001 < *p* < 0.01, and ***p < 0.001.

To determine PSMP chemo-attracted monocytes in a CCR2-dependent manner, we performed adoptive transfer of CD11b^+^Ly6C^hi^ bone marrow cells from the wild type mice (CD45.1) to CCR2 KO mice (CD45.2) (Fig. 2A). The cells were administered intravenously into CCR2 KO recipients coincident with injection of PSMP protein into the abdominal cavities. At 48 h after PSMP injection, the peritoneal cells were harvested and analyzed by flow cytometry. As seen in Fig. 2B,C, the CCR2^+^cells were efficiently recruited by PSMP into the abdominal cavities. The CD45.1^+^Ly6C^hi^CCR2^+^ monocytes and CD45.1^+^CD11b^+^F4/80^+^ macrophages recruited by PSMP into the abdominal cavities in CCR2 KO mice (CD45.2) were significantly more than solvent control group (Fig. 2B–E). These results indicated that the effects of PSMP on the monocytes and macrophages were dependent on CCR2, which was consistent with the PSMP function *in vitro* ^23^.

### PSMP is expressed in human colonic inflammation tissues and adenocarcinomas and significantly up-regulated in mouse DSS-induced colitis tissues

Prior to colonic tumorigenesis, related inflammation occurs in colon tissue^24, 25^. A series of complex reactions occur between immune cells and colonic tissue cells in which different types of bacteria, cytokines, and chemokines are widely involved^26, 27^.Thus, the pro-tumorigenic colonic tissue (colitis) is strongly related to colon tumors.

**Figure 3:**
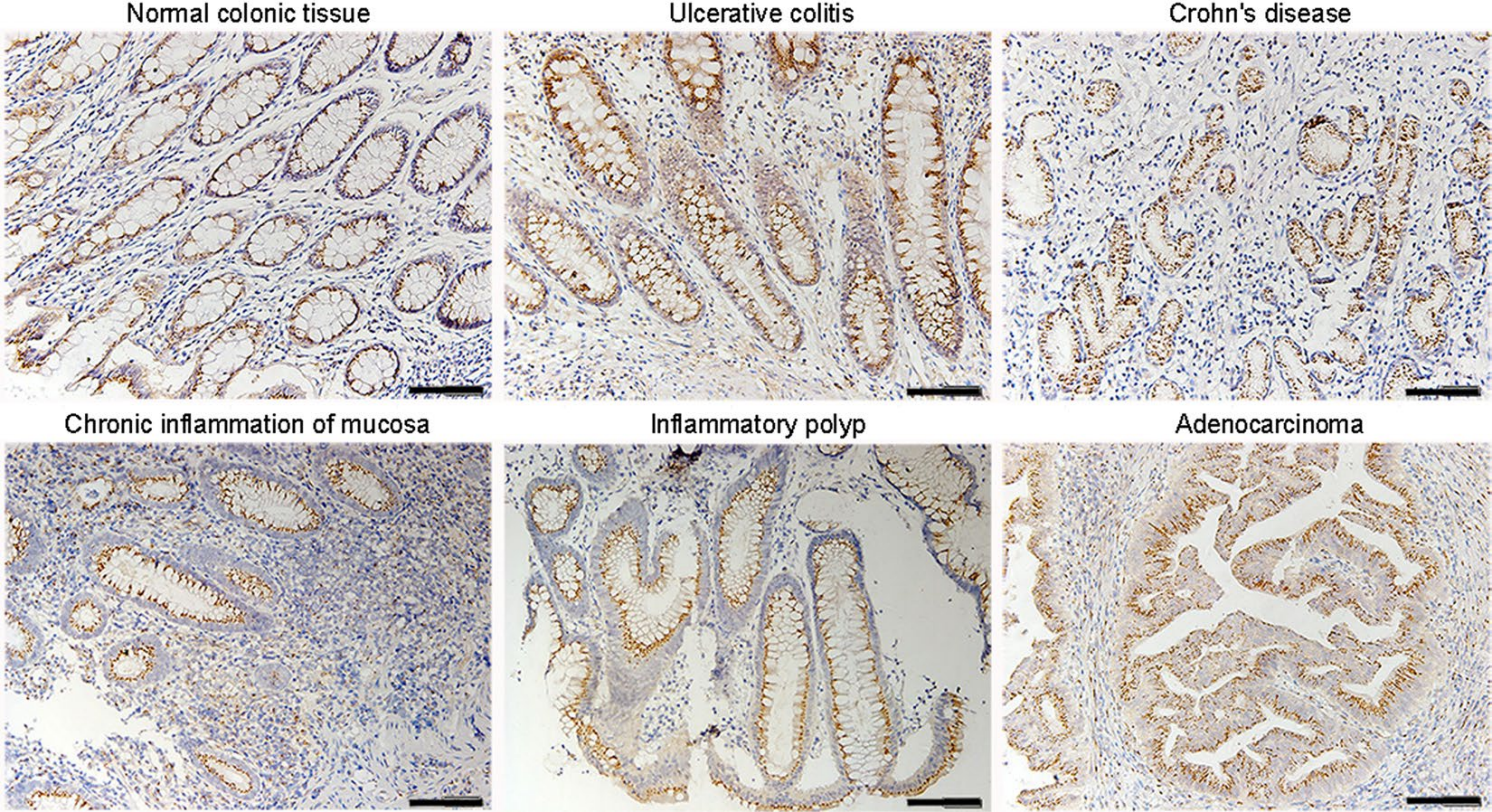
PSMP was expressed in the colonic inflammatory and adenocarcinoma tissues. PSMP expression was detected in the normal colonic, ulcerative colitis, Crohn’s disease, chronic inflammation, inflammatory polyp and adenocarcinoma via IHC with rabbit anti-PSMP antibody (5 μg/mL), scale bar = 100 μm. The colon specimens of each indicated disease were from 4~10 different patients.

As shown in Fig. 3, we found that PSMP was expressed in ulcerative colitis, Crohn’s disease, adenocarcinoma and inflammatory polyp tissues. A colonic tissue microarray was employed. PSMP antibody was used in immunohistochemistry (IHC) for the human tissue microarray.

Based on the indications demonstrated in the results of Fig. 3, a colitis mouse model was employed to further confirm the relationship between PSMP and colitis. The DSS-induced colitis mouse model has been widely used to study the mechanisms of colitis and tumorigenesis, which are similar to those of human ulcerative colitis^28, 29^. The expression of PSMP was detected in the mouse DSS-induced colitis tissue at the mRNA and protein levels. The results revealed significantly higher PSMP expression in the colonic colitis epithelial tissue compared to the normal colonic tissue (Fig. S1A–D).

### Colonic overexpression of PSMP *in situ* chemo-attracts Ly6C^hi^ monocytes in a CCR2-dependent manner

To test the effects of PSMP, we detected the immune cells that were chemo-attracted by the overexpression of PSMP *in situ* in colonic cells in the absence of DSS-induced colitis.

**Figure 4:**
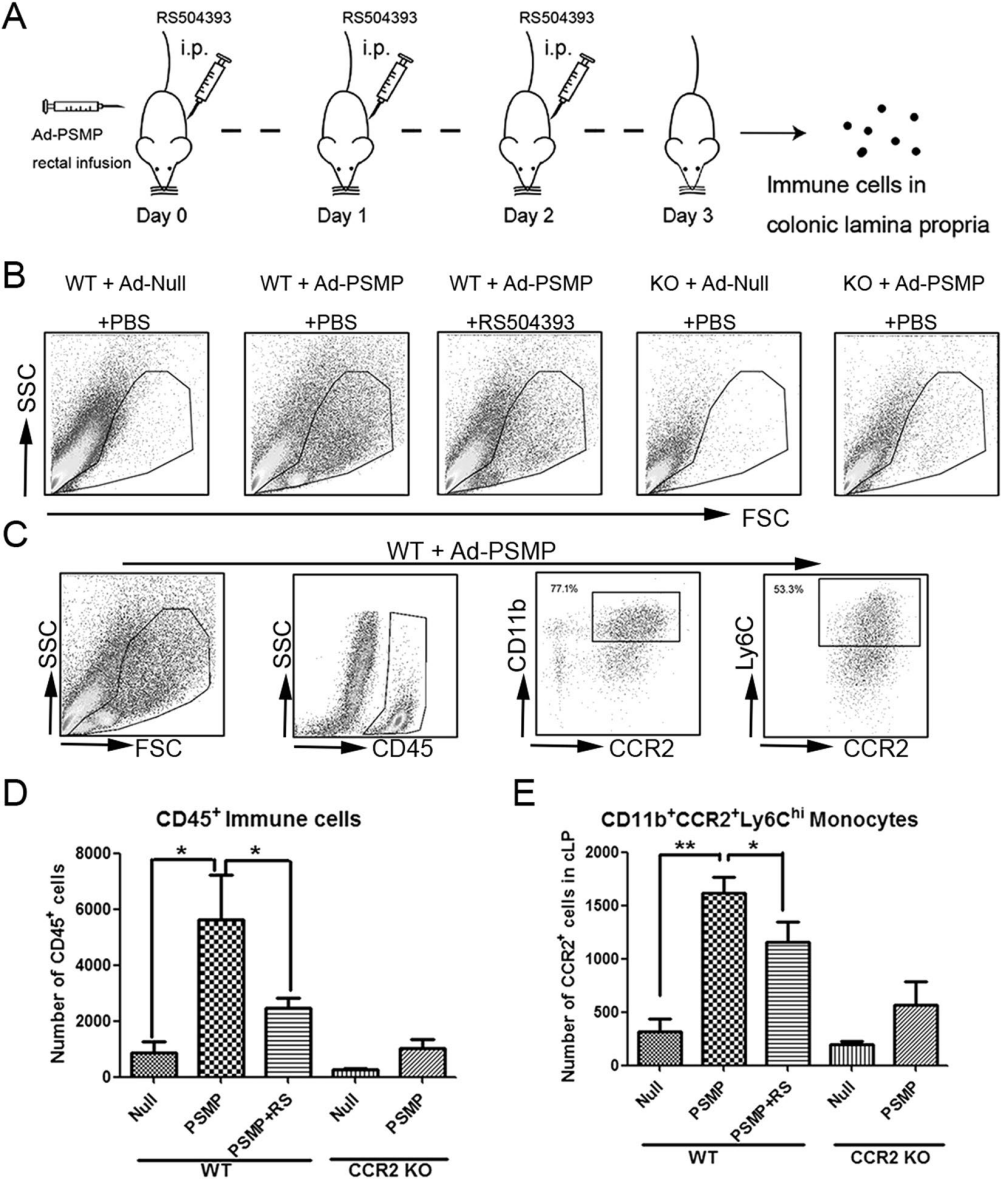
PSMP expression *in situ* in the colon chemo-attracted Ly6C^hi^CCR2^+^ monocytes in a CCR2-dependent manner. (**A**) After the wild type or CCR2 KO mice colons were infected with Ad-Null or Ad-PSMP *in situ* for 72 h, respectively, the immune cells that had infiltrated the lamina propria of the Ad-Null group, Ad-PSMP group and RS504393-treated Ad-PSMP group were detected by flow cytometry; And RS504393 was administrated by *i*.*p*. at the indicated time. (**B**) The immune cells infiltrated into the lamina propria of the five groups were analyzed by flow cytometry. (**C**) The main subset of the infiltrated immune cells in the Ad-PSMP wild type mice was analyzed by flow cytometry with the antibodies for CD45, CD11b, CCR2 and Ly6C, and the percentages of the CD11b^+^CCR2^+^ cells and CD11b^+^CCR2^+^Ly6C^hi^ cells among the total immune cells (CD45^+^) were quantified. (**D**) and (**E**) The total immune cell (CD45^+^) and monocyte (CD11b^+^CCR2^+^Ly6C^hi^) infiltrations in the lamina propria of the five groups were statistically analyzed. Data were representative of at least three independent experiments with five mice per group. *0.01 < *p* < 0.05, **0.001 < *p* < 0.01, and ***p < 0.001.

Following the overexpression of PSMP *in situ* by the Ad-PSMP-infected mucosa, the immune cells that accumulated in the lamina propria were analyzed with flow cytometry (Fig. 4A,B). First, the immune cells (CD45^+^) in the lamina propria of the PSMP up-regulated group obviously exceeded those in the control group (Fig. 4D), which indicated that PSMP could chemo-attract immune cells to the lamina propria. Additionally, the PSMP up-regulated group treated with RS504393 (intraperitoneally injected), which is an antagonist of CCR2 that is widely used *in vivo*, displayed decreased immune cell accumulation in the lamina propria (Fig. 4B–D), which indicated that PSMP chemo-attracted immune cells through CCR2.

Furthermore, the CD11b^+^CCR2^+^Ly6C^+^ monocytes primarily contributed to immune cell accumulation via PSMP-induced chemotaxis (Fig. 4B). The up-regulation of the expression of PSMP significantly elevated the numbers of CD11b^+^CCR2^+^Ly6C^+^ monocytes in the lamina propria (Fig. 4E), and the accumulation of CD11b^+^CCR2^+^Ly6C^+^ monocytes was reduced when the PSMP up-regulated group was treated with RS504393 (Fig. 4E). Regarding neutrophils in the lamina propria, the up-regulation of the expression of PSMP was unable to increase their number (data not shown). In parallel, the CCR2 KO mice were employed for PSMP expression in the mucosa *in situ*, and PSMP was not able to significantly elevate the immune cell or monocyte number in the lamina propria of the CCR2 KO mice (Fig. 4B–E), which indicated that PSMP was unable to chemo-attract the CCR2^−^ immune cells.

The results indicated that the chemotactic ability of PSMP was CCR2-dependent and PSMP directly chemo-attracted CCR2^+^ monocytes in a non-inflammatory environment.

### The up/down-regulation of PSMP expression in the DSS colitis model significantly influences colitis

According to the research discussed above, the expression of PSMP can be up-regulated in colitis tissue. To truly understand the importance of the up-regulation of PSMP in colonic tissue, the expression of PSMP was up/down-regulated artificially with adenovirus or a neutralizing antibody of PSMP.

PSMP adenovirus was used to infect mouse colonic cells via colon lavage. PSMP was successfully overexpressed in the mouse colonic tissue as detected with CBA of the colon homogenate. One day before mouse colitis was induced by DSS, Ad-Null or Ad-PSMP adenoviruses infected the colonic mucosa via colon lavage. The colon length of the group in PSMP up-regulation via Ad-PSMP was significantly shorter than that of the Ad-Null infected group (Fig. S2A,B) and the Ad-PSMP group showed a more serious body weight loss than the Ad-Null group (Fig. S2C). The HE staining and scoring of mouse colonic tissues showed that the Ad-PSMP had a more serious epithelial injury and inflammatory cell infiltration (Fig. S2D,E). These results revealed that the up-regulation of PSMP expression enhanced DSS-induced mouse colitis.

Human PSMP has a 91.4% amino acid sequence identity with mouse PSMP. Both the mouse and human PSMP can chemo-attract mouse macrophages in a CCR2-dependent manner. The PSMP-neutralizing antibody 3D5 can also recognize and neutralize the mouse PSMP and its chemotactic ability (data not shown). Besides, anti-PSMP antibody 3D5 was specificity to neutralize PSMP (Fig. S3).

It was also necessary to determine whether the down-regulation of PSMP expression affected colitis development. The PSMP-neutralizing antibody 3D5 was employed in this experiment. The 3D5 or mIgG (5 mg/kg) was intraperitoneally injected into the mice on days 0, 2, 4, 6 after starting DSS oral administration.

**Figure 5:**
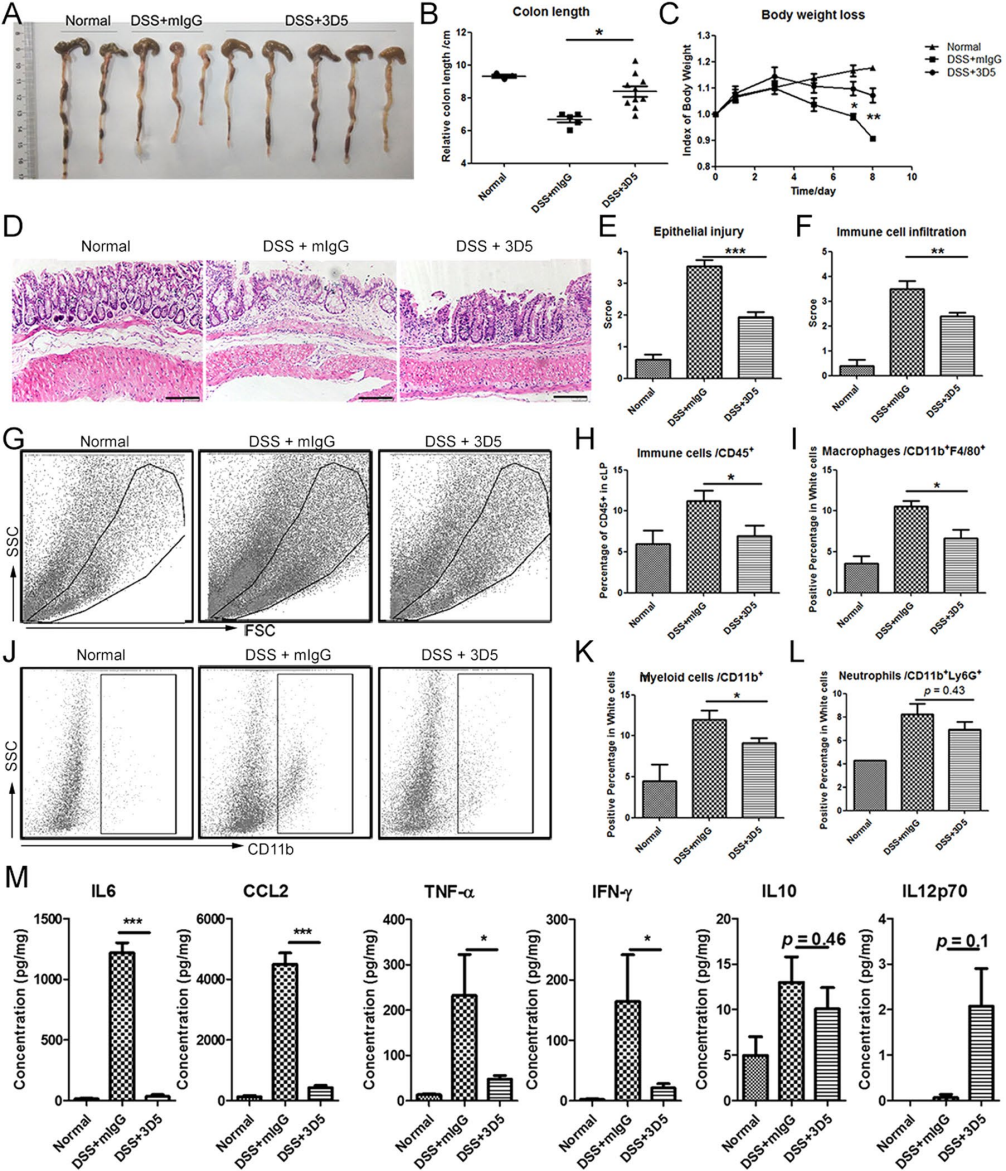
The neutralizing antibody of PSMP mollified DSS-induced colitis via its effects on the monocytes/macrophages and IL-6, TNF-α, CCL2, IFN-γ. Based on the DSS-induced colitis, PSMP neutralizing antibody 3D5 (5 mg/kg) or mouse immunoglobulin G (mIgG) as a negative control were peritoneal injected every other day. (**A**) The colons were separated from the normal, DSS-induced colitis and 3D5-treated DSS-induced colitis groups. (**B**) and (**C**) The lengths of the colons and the body weight of the three groups were measured and statistically analyzed. (**D**) The colonic tissue biopsies from the three groups were stained with HE. Scale bar = 100 μm. (**E**) and (**F**) The colonic inflammatory score of the three groups were statistically analyzed based on the conditions of the colonic epithelial injuries and immune cell infiltration of cLP. (**G**) The immune cells that infiltrated the cLP in the three groups were separated and analyzed by flow cytometry. The Gate in the FSC-SSC graph displayed the distribution of immune cells. (**H**) The immune cell (CD45^+^) infiltrations in the three groups were statistically analyzed. (**I**) The percentages of macrophages (F4/80^+^) among all of the immune cells in the cLP of the three groups were statistically analyzed. (**J**–**L**) The myeloid-derived cells (CD11b^+^) and granulocytes (Ly6G^+^) in the total immune cells of the three groups’ cLP were displayed in the CD11b-SSC graph and were statistically analyzed. (**M**) The inflammatory cytokines IL-6, TNF-α, CCL2, IFN-γ, IL-10 and IL-12p70 were detected in the colonic tissue homogenates by CBA, and the concentrations of these cytokines in the colon tissues of the three groups were statistically analyzed. Data were representative of at least three independent experiments with five mice per group. *0.01 < *p* < 0.05, **0.001 < *p* < 0.01, and ***p < 0.001.

The 3D5-treated group exhibited longer colons than the mIgG group, which were indicative of less extensive colitis (Fig. 5A,B). In parallel, the body weights were acquired and revealed that the weight loss of the 3D5-treated group was significantly less than that of the mIgG group on days 6 and 7 (Fig. 5C). Colon tissue biopsies of the three groups were HE stained, and evaluated for the conditions of colonic epithelial destruction and immune cell infiltration (Fig. 5D–F). The results revealed that 3D5 reduced the extent of DSS-induced colitis.

Immune cell infiltration is an important aspect of colitis^30^. When colitis occurs, numerous inflammatory cells accumulate in the colonic lamina propria^31^. Flow cytometry was used to detect inflammatory cells infiltrated into the lamina propria, and inflammatory cells (CD45^+^) accumulated on a large scale in the DSS-induced colitis with mIgG-treated group compared with the normal group (Fig. 5G,H). The lamina propria of the 3D5-treated group contained significantly fewer CD45^+^ inflammatory cells than that of the mIgG-treated group (Fig. 5H), which was consistent with the HE staining biopsy observations. After 3D5 treatment, fewer macrophages (CD11b^+^F4/80^+^) accumulated in the lamina propria, but neutrophils (CD11b^+^Ly6G^+^) were not significantly affected (Fig. 5I–L). Therefore, the therapeutic effect of 3D5 on colitis may result from reducing monocyte/macrophage accumulation; in other words, PSMP might participate in colitis via the chemo-attraction of monocytes.

CD11b^+^Ly6C^+^monocytes are recognized as the classical pro-inflammatory monocytes and transform into inflammatory macrophages upon arrival at colitic lamina propria^32^. The cytokines secreted by these macrophages, including as IL-6, CCL2 and TNF-α, promote inflammation and help to invoke many types of inflammatory cells. These related cytokines were detected with CBA in the colon homogenates of the three groups. IL-6, CCL2, TNF-α, and IFN-γ in the mIgG-treated colitis group were extremely elevated and significantly decreased in the 3D5-treated colitis group (Fig. 5M). These cytokine detection results further support the speculation that PSMP participates in colitis and the therapeutic effect of PSMP-neutralizing antibody 3D5 on colitis.

### The expression of PSMP is up-regulated in the colonic epithelial cells in the initial stage of the DSS colitis model

The mechanism of colitis is not fully understood. It is known that epithelial cells and resident immune cells activate and release inflammatory signals following the microbes exposed to injured colonic mucosa^33^. Subsequently, inflammatory cells, including neutrophils, monocytes, and activated T cells, arrive under the guidance of inflammatory signals and establish an inflammatory environment^34^. Monocytes have been proved to be essential and important in colitis^35^.

The above results demonstrate that PSMP can act as the chemokine on monocytes. However, CCL2 is also a well-known chemokine for monocytes, and CCL2 can be secreted by monocytes and macrophages^36^. Therefore, we sought to determine whether and when PSMP or CCL2 are up-regulated during colitis.

**Figure 6:**
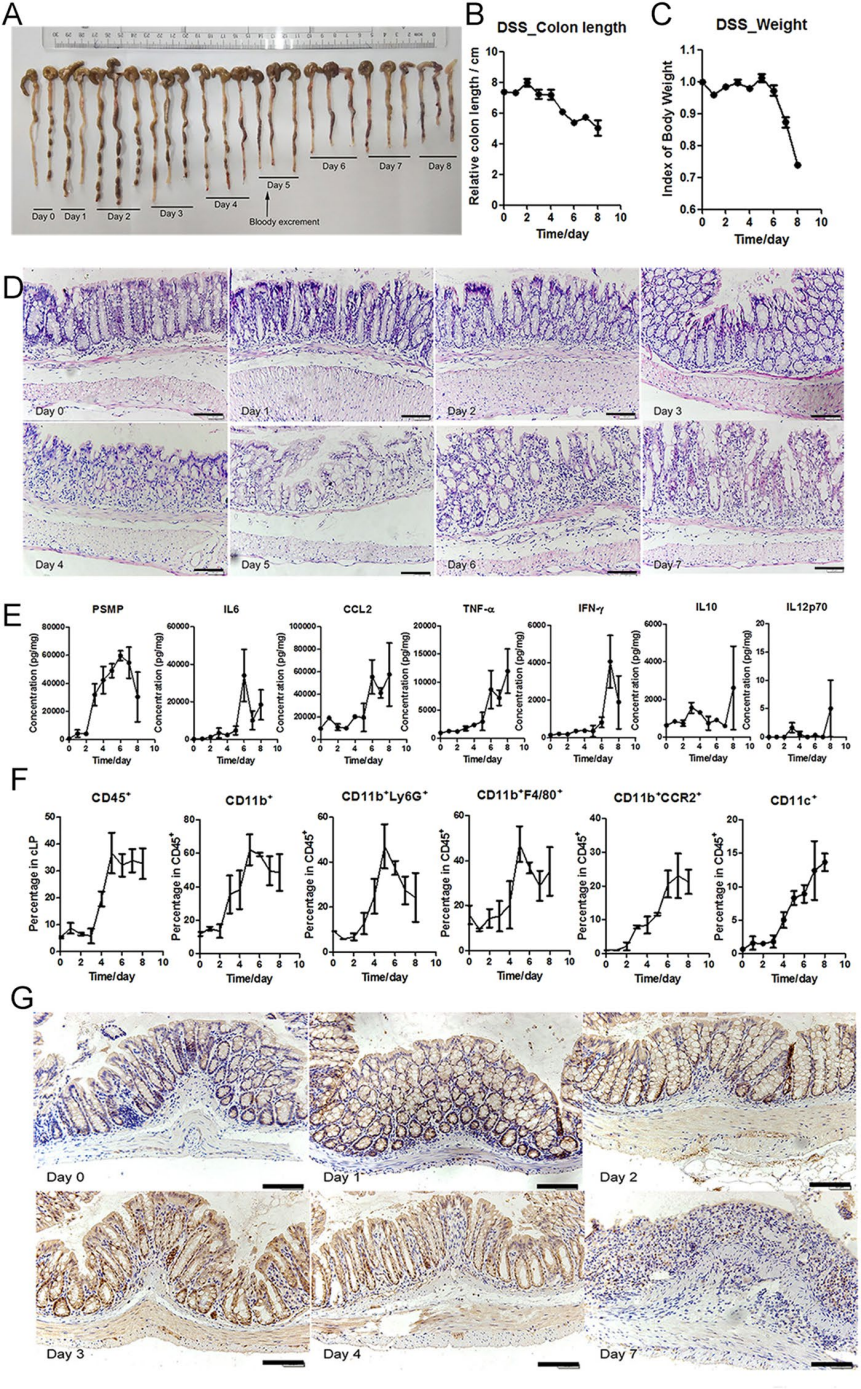
PSMP expression was up-regulated in the initial stage of DSS-induced colitis. (**A**) The mouse colons were separated each day during colitis induction, and on the fifth day of colitis, bloody excrement was observed. (**B**) The colon lengths were measured and statistically analyzed each day during the induction of colitis. (**C**) The body weights of the mice were measured and statistically analyzed each day during the induction of colitis. (**D**) The colonic tissue biopsies were stained with HE. (**E**) The concentrations of PSMP, IL-6, TNF-α, CCL2, IFN-γ, IL-10, and IL-12p70 in the DSS colitis tissue homogenates over time were detected by CBA. (**F**) The percentages of immune cells (CD45^+^) among the cLP cells and the percentages of myeloid-derived cells (CD11b^+^), granulocytes (CD11b^+^Ly6G^+^), macrophages (CD11b^+^F4/80^+^), CCR2^+^ myeloid-derived cells, and CD11c^+^ among the CD45^+^ immune cells over time detected by flow cytometry. (**G**) The time course of PSMP expression in the colonic tissue biopsies by PSMP IHC staining. Scale bar = 100 μm. Data were representative of at least two independent experiments with three mice per group. *0.01 < *p* < 0.05, **0.001 < *p* < 0.01, and ***p < 0.001.

To achieve this aim, a time-course study of a DSS-induced colitis model was established. On the fifth day in this model, the body weights of the mice were significantly reduced, the colons were obviously shorter, bloody stools appeared (Fig. 6A–C) and the epithelial injury and inflammation cell infiltration got more serious (Fig. 6D). Simultaneously, cytokines, including PSMP, IL-6, CCL2, TNF-α, IFN-γ and IL-10 in the colon homogenates were measured with CBA in the time-course study of colitis. IL-6, CCL2 and TNF-α displayed significant increases on the sixth day and IFN-γ was elevated on the seventh day (Fig. 6E). Interestingly, the protein expression of PSMP was up-regulated on the third day in this colitis model (Fig. 6E), which paralleled the elevation of the CD11b^+^CCR2^+^ monocytes (Fig. 6F). Additionally, IHC of the colon tissue biopsies of this time-course model revealed that PSMP was highly expressed by the colonic epithelial cells (Fig. 6G).

Total immune cells (CD45^+^), myeloid-derived cells (CD11b^+^), monocytes (CD11b^+^CCR2^+^), macrophages (CD11b^+^F4/80^+^), neutrophils (CD11b^+^Ly6G^+^), and CD11c^+^ in the colonic lamina propria were detected each day during the induction of colitis. Total immune cells (CD45^+^), macrophages (CD11b^+^F4/80^+^), neutrophils (CD11b^+^Ly6G^+^) and CD11c^+^ cells exhibited apparent accumulation on the fifth day in this model, which corresponded to the colitis becoming serious on the fifth day (Fig. 6F). Interestingly, the myeloid-derived cells (CD11b^+^) and monocytes (CD11b^+^CCR2^+^) exhibited obvious elevation on the third day (Fig. 6F) that occurred earlier than those of the other immune cells.

Based on these results, PSMP was identified as a chemokine that was expressed by colonic epithelial cells in the initial stages of colitis and promotes inflammation by affecting monocyte accumulation. PSMP expression was up-regulated by colonic epithelial cells before the up-regulation of the expression of important inflammatory cytokines, including CCL2, which indicated that PSMP but not CCL2 acted first to play a key role in the initial phase of colitis in the PSMP-CCR2-monocyte axis.

### PSMP is induced by LPS and MDP in colon epithelial cells and promotes CCL2 expression in M1 macrophages

Pathogen-associated molecular patterns (PAMPs) and pattern recognition receptors (PRRs) have been shown to play a crucial role in triggering immunity. When the balance between microbe-host homeostasis in the intestinal tract is disrupted, CECs will firstly contact microbes and recognize the microbial products, namely PAMPs, via PRRs. The PRRs expressed on CECs, such as TLRs and NLRs, can recognize PAMPs and initiate signaling pathways and cytokine production^37, 38^.

**Figure 7:**
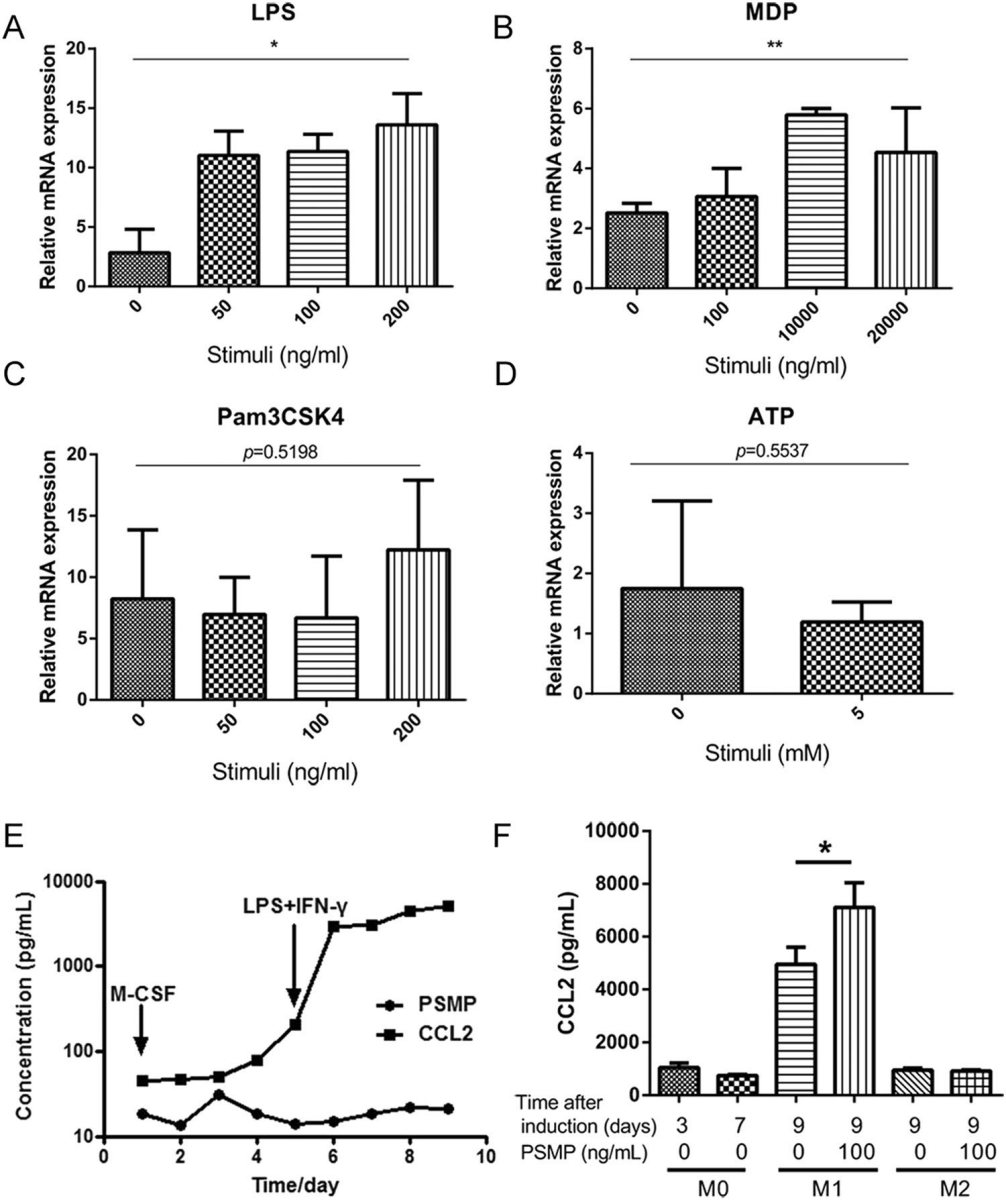
PSMP expression was induced by LPS and MDP in CECs, and PSMP promoted the expression of CCL2 in M1 macrophages. (**A**–**D**) Primary CECs from wild type mice were stimulated with LPS, Pam3CSK4, MDP or ATP for 3 h. PSMP mRNA expression was assessed by real-time PCR. (**E**) During the monocyte-macrophage polarization procession, the PSMP expression in the cell culture medium was detected by CBA and CCL2 expression was detected in parallel by ELISA. (**F**) The CCL2 expression was detected in the medium of M0, M1 and M2 cells with and without PSMP (100 ng/mL) stimulating at the indicated time by ELISA and statistically analyzed. Data were representative of at least three independent experiments. *0.01 < *p* < 0.05, **0.001 < *p* < 0.01, and ***p < 0.001.

According to our data above, an increased expression of PSMP occurs in the initial stages of colitis and mainly in CECs. To further investigate which signal(s) regulated the expression of PSMP, we isolated and stimulated the CECs with toll-like receptor (TLR)-4 agonist lipopolysaccharide (LPS), nucleotide oligomerization domain-2 (NOD2) agonist muramyl dipeptide (MDP), TLR1/2 agonist Pam3CSK4 or inflammasome agonist ATP. The results showed that LPS or MDP led to an increased expression of PSMP in a dose-dependent manner (Fig. 7A,B), but Pam3CSK4 or ATP did not lead to a similar PSMP enhancement (Fig. 7C,D). It suggested that TLR4 and NOD2 pathways were important for the expression of PSMP by CECs.

Because we found that the up-regulated expression of PSMP was earlier than CCL2 in DSS-induced mouse colitis, we explored whether PSMP regulated CCL2 expression in monocytes or macrophages. It is well known that CCL2 is primarily secreted by monocytes, macrophages, and dendritic cells^18–20^. M1 macrophages are known as classically activated macrophages and secrete pro-inflammatory cytokines and chemokines attracting other types of immune cells and integrating/orchestrating the immune response. During a M0-M1 macrophage polarization procession, CCL2 expression was up-regulated but not PSMP (Fig. 7E). We hypothesized that PSMP was up-regulated in the CECs and paracrine to promote the CCL2 expression in M1 macrophages in colitis. To explore whether PSMP regulated the expression of CCL2, the experiments *in vitro* were designed that PSMP stimulated the M0-M1 or M0-M2 macrophages, and then secreted CCL2 was detected by ELISA. The results suggested that CCL2 was significantly higher expressed on the M1 macrophages under the stimulation of PSMP (Fig. 7F).

## Discussion

PSMP has been reported to be a chemokine-like protein, and its chemotactic receptor has been identified as CCR2 *in vitro* ^23^. However, the relationship between PSMP and disease remains unclear. In the present research, we focused on understanding the role of PSMP in colitis.

First, the relationship between PSMP expression and ulcerative colitis was explored. PSMP was expressed in human colitic tissues and significantly up-regulated in DSS-induced mouse colitis. Interestingly, PSMP increases were observed in the initial stage in the DSS-induced colitis model; at this time, CCL2, IL-6 and TNF-α were still expressed at low level. Moreover, immunohistochemistry results showed that PSMP was mainly expressed in the colonic epithelium. PAMPs and PRRs have been shown to play a crucial role in triggering immunity^37, 38^. Thus, we explored which signal(s) regulated PSMP. Our data demonstrated that the up-regulated expression of PSMP was induced by PAMPs in CECs, which can explain the PSMP increase in the initial stage of colitis and further support PSMP expressed in the colonic epithelium. In this study, PSMP was not found to be expressed by the monocytes/macrophages or immune cells accumulation area. A main source of CCL2 expression has been reported to be monocytes and “classical pro-inflammatory” macrophages^18–20^. During a M0-M1 macrophage polarization procession, our data showed that CCL2 expression was up-regulated but PSMP kept at low level. Because we found that the up-regulated expression of PSMP in DSS-induced mouse colitis was earlier than CCL2, we hypothesized that PSMP was up-regulated in the CECs and paracrine to promote the CCL2 expression in M1 macrophages in colitis. Thus, we further explored whether PSMP regulated CCL2. Our data demonstrated that CCL2 was significantly higher expressed on the pro-inflammatory M1 macrophages under the stimulation of PSMP. These results suggest that PSMP is expressed by colonic epithelial cells in the initial stages of colitis before the up-regulation of the expression of important inflammatory cytokines and further promotes production of CCL2 by pro-inflammatory macrophages in colitis, which indicates that PSMP but not CCL2 might act first to play a key role in the initial phase of colitis.

Colitis-related innate immunity has come to be widely studied by many teams^39, 40^. Innate immunity is strongly connected with mucosal damage, repair and tumorigenesis^41, 42^. Many types of immune cells, including macrophages^8^, DCs^43^, Treg cells^44^, T-helper cells^45^, and natural killer T cells^46^, have been reported to participate in colitis-related innate immunity. The chemokines that help the precursors of these immune cells to migrate into the mucosa have been little investigated. The precursors of macrophages and DCs in both homeostasis and inflammation have been found to be monocytes from the circulation^47^. Monocytes in the circulation have been classified into two sub-groups^48^. The first is the Ly6C^hi^CCR2^+^CD62L^+^CX_3_CR1^mid^ mouse monocyte subset and the human counterpart CD14^+^ cells that are generated from bone marrow and whose precursors are monocyte-dendritic cell progenitors and common monocyte progenitors^49^. CCR2 has been proved to be essential for the release of this subset from the bone marrow^14–17^, and this subset is recognized as the classical monocytes that are the precursors of the peripheral mononuclear phagocytes^50^. The other subset is the Ly6C^low^CD43^+^CX_3_CR1^hi^ mouse monocyte subset and their CD14^low^CD16^+^ human counterparts that are generated from the circulation and whose precursors are Ly6C^hi^ monocytes^51^. This monocyte subset resides in the lumen and monitors endothelial integrity^51^. The Ly6C^hi^CCR2^+^CX_3_CR1^mid^ monocytes are the classical inflammatory monocytes that exist in the peripheral blood, and once colitis has occurred, this subset is chemo-attracted and participates in inflammation^9, 10, 12^. Although the important role of CCR2 in monocytes migrating into the lamina propria and promoting colitis has been proved^14–17^, the chemokine that controls the earliest-arriving monocytes from the circulation to the lamina propria remains to be discovered. One team found that similar colitis can be induced in CCL2 KO mice and wild types, which indicated that the effects of CCR2 in colitis are independent of CCL2^21^.

Further, our data in this study demonstrated that PSMP overexpression enhanced DSS-induced mouse colitis and a neutralizing antibody of PSMP mollified colitis by reducing macrophage accumulation, suggesting that PSMP is a key protein in ulcerative colitis. The chemotactic potential of PSMP for monocytes was further demonstrated in a non-inflammatory colon and abdominal cavity *in situ*. We found that effects of PSMP on colitis were primarily dependent on its influence on monocytes/macrophages and that chemotactic progression relied on CCR2. The results from the CCR2 KO mice, adoptive transfer of CD11b^+^Ly6C^hi^ bone marrow cells and CCR2 antagonist assay revealed that PSMP chemotactic effects on Ly6C^hi^ monocytes depend on CCR2. Therefore, our results revealed that PSMP is a chemokine that triggers the earlier-arriving Ly6C^hi^CCR2^+^ monocyte migration from the circulation into the tissue that depends on CCR2 and further promotes colitis.

CCL2 is a most well-known ligand of CCR2, whose ligands also include CCL7 and CCL8. Our previous study demonstrated that PSMP acts as a new ligand of CCR2. The affinity between PSMP and CCR2 is found to be comparable to that between CCL2 and CCR2^23^. In this study, PSMP increases were observed in the initial stage in the DSS-induced colitis model; at this time, CCL2, IL-6 and TNF-α were still expressed at low level. Besides CCL2, we also detected other ligands of CCR2, namely CCL7 and CCL8, and found the expression of PSMP at mRNA levels was up-regulated on the second day prior to CCL2 and CCL7 increases, while expression of CCL8 remained at low levels (Fig. S4). In addition, a study demonstrated that there were significantly enhanced gene and/or protein expression of chemokines CCL2 and CCL7 in colitis patients^52^. These results indicated that CCL2 and CCL7 might also participate in DSS-induced colitis, but PSMP with a more important role in the initial stage of colitis differed from other conventional ligands for CCR2. We speculated that the redundancy of PSMP, CCL2 and CCL7 in colitis might result in that CCR2 but not the CCL2 is vital to the progression of colitis. Thus, further investigation is necessary to determine the synergistic role of PSMP, CCL2 and CCL7 in colitis as well as determining whether they represent the therapeutic targets.

Regarding tumorigenesis, there are reports that CCR2 but not CCL2 expression can be up-regulated during azoxymethane (AOM)/DSS induced colonic tumorigenesis^53^. PSMP has also been detected at higher level in the serum of AOM/DSS mice compared with normal mice, and a neutralizing antibody of PSMP can dramatically suppress colonic tumorigenesis (unpublished data), which indicates that PSMP might also play a vital role in colonic tumorigenesis.

**Figure 8:**
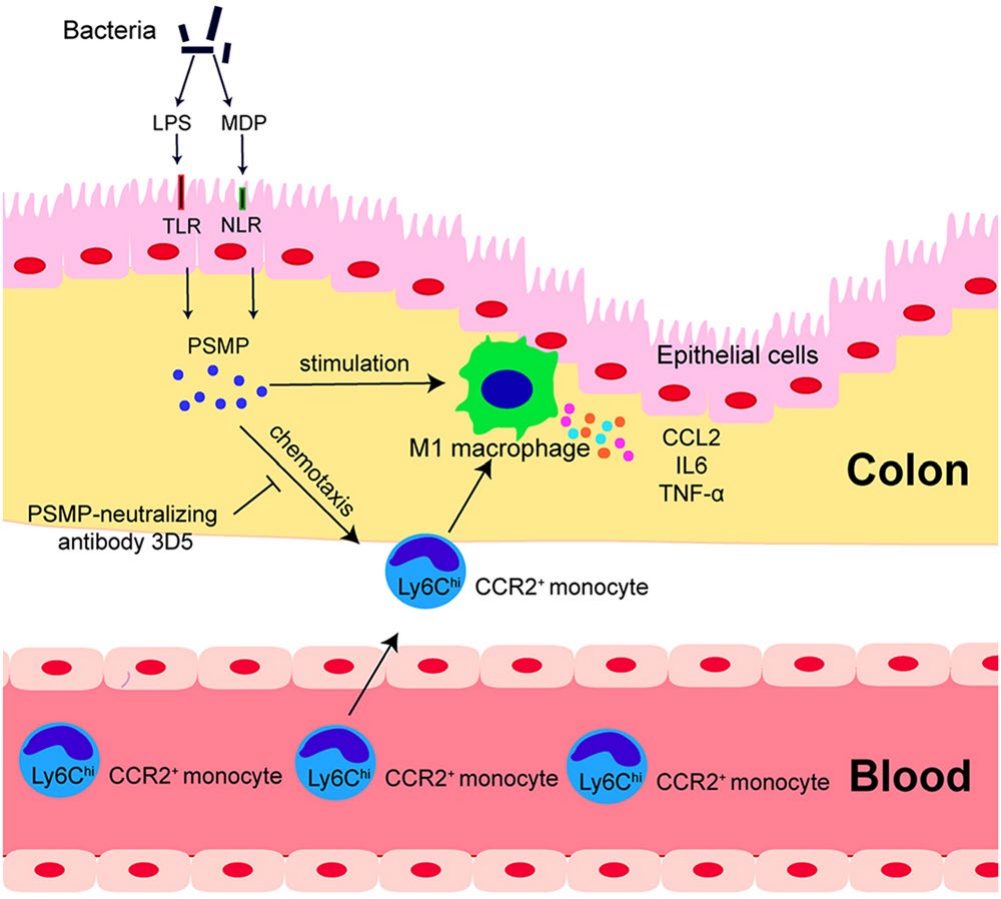
Summary diagram of the mechanisms by which the PSMP-CCR2 system affects colitis. The bacterial products LPS and MDP stimulate colonic epithelial cells to produce PSMP at the early stage of colitis prior to IL-6, TNF-α and CCL2 up-regulated expression. PSMP recruites Ly6C^hi^CCR2^+^ monocytes from blood to injuried colon via a CCR2-dependment manner and promotes M1 macrophages to produce CCL2. Secreted CCL2 further recruites Ly6C^hi^CCR2^+^ monocytes from blood to inflammation sites and enlarges the reaction to promote colitis in turn. A neutralizing antibody of PSMP can mollify colitis by reducing macrophage accumulation.

In summary, the data from this study show for the first time that PSMP plays a vital role in promoting DSS colitis. PSMP is expressed in human colitis tissues and significantly up-regulated DSS-induced mouse colitis. PSMP is up-regulated in the initial stage prior to IL-6, TNF-α and CCL2 up-regulated expression in DSS colitis and promote the M1 macrophages to produce CCL2. LPS or MDP can induce PSMP up-regulation in the colonic epithelial cells. PSMP chemo-attracts Ly6C^hi^ monocytes in a CCR2 dependent manner. PSMP might trigger the earlier-arriving Ly6C^hi^CCR2^+^ monocyte migration from the circulation into the tissue and synergize with CCL2 and CCL7 to promote colitis (Fig. 8). Targeting the CCR2-PSMP axis might turn out to be a promising new strategy for the treatment of intestinal inflammatory diseases.

## Materials and Methods

### Reagents, protein and antibody

RPMI 1640 medium and fetal bovine serum (FBS) were from Life Technologies (Carlsbad). PSMP was obtained as previously described^23^. DSS (molecular weight 36 000–50 000 Da) was purchased from MP Biomedicals LLC (Solon). Mouse anti-CCR2, RS504393 was purchased from Tocris Bioscience (Ellisville).

Antibodies for flow cytometry were from BioLegend. The adenovirus with the PSMP gene (Ad-PSMP) and the empty adenovirus (Ad-Null) were purified by SinogenoMax (Beijing, China). The neutralizing antibody for PSMP (3D5) was obtained and purified as previous protocol^23^. The rabbit polyclonal antibody for PSMP was purchased from Sigma (Sigma-Aldrich).

### Animals

The 6-week-old male C57BL/6 mice and CD45.1 wild type mice were purchased from Peking Experimental Animal Center (Beijing, China). The 6-week-old male CCR2 knockout mice on C57BL/6 background were obtained from Professor Yu Zhang (Department of Immunology, Peking University Health Science Center). The mice were raised in a specific pathogen-free animal facility. All animal experiments were carried out according to the Guidelines for the Care and Use of Laboratory Animals and were approved by the Ethics Committee of Peking University Health Science Center.

### Abdominal *in situ* chemotaxis assay

The wild type or CCR2 KO mice were intraperitoneally injected with or without PSMP (40 μg per mouse) dissolved in Pluronic F127 (30%, w/v, P2443-250G) (Sigma-Aldrich). The mice were sacrificed after 48 h. The abdominal immune cells were collected and detected.

### Adoptive transfer assay

The bone marrow cells from the CD45.1 wild type mice were labeled with CD11b-PE and Ly6C-PerCP-Cy5.5 and double-positive (CD11b^+^Ly6C^+^) cells were sorted with a flow cytometer. The sorted cells of the CD45.1 wild type mice (1 × 10^7^cells per mouse) in PBS were intravenously injected into the CD45.2 CCR2 KO mice. The mice were sacrificed after 48 h, and the abdominal immune cells were collected and detected via flow cytometry.

### Colon *in situ* chemotaxis assay

The colons of wild type or CCR2 KO mice were anally administered Ad-PSMP or negative control Ad-Null at a dose of 1 × 10^9^ pfu (50 μL) per mouse. The wild type mice administered Ad-PSMP were intraperitoneally injected with RS504393/phosphate buffer saline (PBS) (25 μL/500 μL per mouse) or with PBS as the negative control. The mice were sacrificed after 72 h. The infiltrated immune cells in colonic lamina propria were detected.

### DSS-induced colitis

The mice were provided free access to food and water in a specific pathogen-free animal facility. Acute colitis was induced by administration of 3% (w/v) DSS ad libitum for a week. The mice were weighed and monitored for diarrhea and blood in their stools. In the DSS time-course model, three mice were killed each day during the oral administration. The cytokines and infiltrated cells were detected.

### Isolation and stimulation of colon epithelial cells and colonic lamina propria cells

The isolation of colon epithelial cells (CECs) and colonic lamina propria (cLP) cells have been described previously^54^. Briefly, colons were washed off fecal contents and cut into 1.5cm long pieces. Colon pieces first digested with 5 mmol/L EDTA and 1 mmol/L dithiothreitol (DTT) for 30 min at 37 °C. The cell suspension was passed over a 200 μm coarse mesh and centrifugated. The cell pellet was harvested as CECs. Then rest colon pieces were digested with 1 mg/ml type IV collagenase (Roche) and 150 U/ml DNase I (Sigma-Aldrich). The digested cell suspension was passed over a 200 μm coarse mesh and centrifugated. The cell pellet was harvested as the cells in cLP. Primary CECs were plated in 6-well dishes prior to stimulation with LPS (Sigma-Aldrich), Pam3CSK4 (Invivogen), MDP (Invivogen), or ATP (Invitrogen) for 3 h and assessed by real-time PCR. The cells in cLP were stained with antibody for flow cytometric analysis.

### Flow cytometric analysis

Peripheral blood mononuclear cells, peritoneal cells or lamina propria cells were taken and blocked in 5% fetal bovine serum for 20 min and then stained with CD45-FITC, CD11b-PE, CCR2-APC, Ly6C-PerCP Cy5.5, Ly6G-PerCP Cy5.5, F4/80-APC, or/and CD11c-APC (BioLegend) at 4 °C for 30 min. Data were acquired from FACS caliber (BD Biosciences) and analyzed using FlowJo 7.6 (TreeStar).

### Real-time polymerase chain reaction (PCR)

The tissue or cells were homogenized in Trizol (Invitrogen). Reverse transcription (RT) reactions were performed by using the RT MasterMix system (ABM Inc.). Real-time PCR was carried out using SYBR Green PCR mix (ABM Inc.). All gene expressions were normalized to that of *Gapdh*. The primers of mouse *Psmp* were 5′-CTGTGACACGGCTCAGCATC-3′ (forward) and 5′-ATGGGCAAGCCTTTAGCTGG-3′ (reverse). The primers of mouse *Gapdh* were 5′-CGGAGTCAACGGATTTGGTCGTAT-3′ (forward) and 5′-AGCCTTCTCCATGGTGGTGAAGAC-3′ (reverse).

### Cytometric bead assay (CBA)

Microspheres (A37304, Life Technologies) were coated with polyclonal rabbit anti-PSMP antibody according to the manufacturer’s instructions. The coated beads were blocked with 20% FBS for 20 min and then added to the samples for 2 h at room temperature, and the monoclonal anti-PSMP antibodies were then added for 1 h. The mixture was suspended with PE goat anti-mouse antibody (eBiocience) for 30 min at room temperature. The data were acquired by flow cytometry.

The cytokines IL-6, IL-10, CCL2, IFN-γ, TNF-α, and IL-12p70 were detected with a BD CBA mouse inflammation kit (552364, BD Biosciences) according to the instruction manual. Flow cytometry was used to acquire data.

### Histological staining and scoring

Animals were sacrificed and distal colon were fixed in 10% formalin, cut at 4 μm and stained with hematoxylin and eosin (HE). Histological score of colon were assessed as follows^55^.Injured epithelium score:0, normal morphology; 1, loss of goblet cells; 2, loss of goblet cells in large areas; 3, loss of crypts; 4, loss of crypts in large areas. Infiltrated immune cells score: 0, no infiltrate; 1, infiltrate around crypt basis; 2, infiltrate reaching to submucosa; 3, extensive infiltration reaching the muscularis mucosae; 4, infiltration of muscular layer. Pathological change range score: 0, none; 1, 1–25%; 2, 26–50%; 3, 51–75%; 4, 76–100%. The histological score of colitis was calculated as (injured epithelium score + infiltrated immune cells score) × Pathological change range score.

The human tissue microarray containing multiple colon specimens was purchased from Chinese Shanxi Chaoying Biotechnology Co., Ltd. The collection of clinical specimens was approved by the Ethics Committee of Tongxu County People’s Hospital in Henan province. Immunohistochemistry was carried out as previously described^23^.

### Differentiation of monocytes to macrophages *in vitro*

This procession was conducted followed the protocol from previous research^56^. Bone marrow cells were flushed out from the hind legs of wild type mice using DMEM medium. To generate differentiated M0 macrophages, bone marrow-derived macrophages were incubated for 6 d with 10 ng/ml M-CSF (R&D Systems). M0 macrophages were incubated in the presence of either 20ng/ml IFN-γ (R&D Systems) and 100 ng/ml LPS (Sigma-Aldrich) for 24 h to polarize into M1 macrophages or 20 ng/ml IL-4 (R&D Systems) to polarize into M2 macrophages. The expression of PSMP and CCL2 were assessed by CBA or ELISA kit (R&D Systems), respectively. Then M0, M1 and M2 macrophages were stimulated with PSMP at the indicated dose.

### Statistical analysis

The data of 3~6 mice in each group were performed as the means ± SEM using Student’s *t* test with GraphPad Prism program (GraphPad Software). Statistical significance differences between two groups are presented by *0.01 < *p* < 0.05, **0.001 < *p* < 0.01, and ***p < 0.001.

## Electronic supplementary material

Supplementary information
