# Supplementary material for: The PSMP-CCR2 interactions trigger monocyte/macrophage-dependent colitis
Source: Sci Rep. 2017 Jul 11;7:5107. doi: 10.1038/s41598-017-05255-7 (PMC5506041; doi:10.1038/s41598-017-05255-7)
Supplement: Supplementary file 1 — Supplementary information [file 41598_2017_5255_MOESM1_ESM.pdf]

The PSMP-CCR2 interactions trigger monocyte/macrophage-dependent colitis

Xiaolei Pei[1, 3,*], Danfeng Zheng[1,*], Shaoping She[1], Jing Ma[1], Changyuan Guo[4], Xiaoning Mo[2], Yingmei Zhang[1, 2], Quansheng Song[1, 2], Yu Zhang[1], Dalong Ma[1, 2], and Ying Wang[1, 2]

[1]Department of Immunology, School of Basic Medical Sciences, and Key Laboratory of Medical Immunology of Ministry of Health, Peking University Health Science Center, Beijing 100191, P.R.China.

[2]Center for Human Disease Genomics, PekingUniversity, Beijing 100191, P.R.China.

[3]Institute of Hematology and Blood Diseases Hospital, Chinese Academy of Medical Sciences and Peking Union Medical College, 288 Nanjing Road, Tianjin, 300020, P.R.China.

[4]Department of Pathology, Cancer Institute and Hospital, Chinese Academy of Medical Sciences, 17 Panjiayuan South Lane, Chaoyang District, Beijing 100021, P.R.China.

*These authors contributed equally to this paper.

Address correspondence and reprint requests to Ying Wang,38 Xueyuan Road, Beijing 100191, P.R.China. Tel.: +86-10-82802846-5032; Fax: +86-10-82801149; E-mail: yw@bjmu.edu.cn

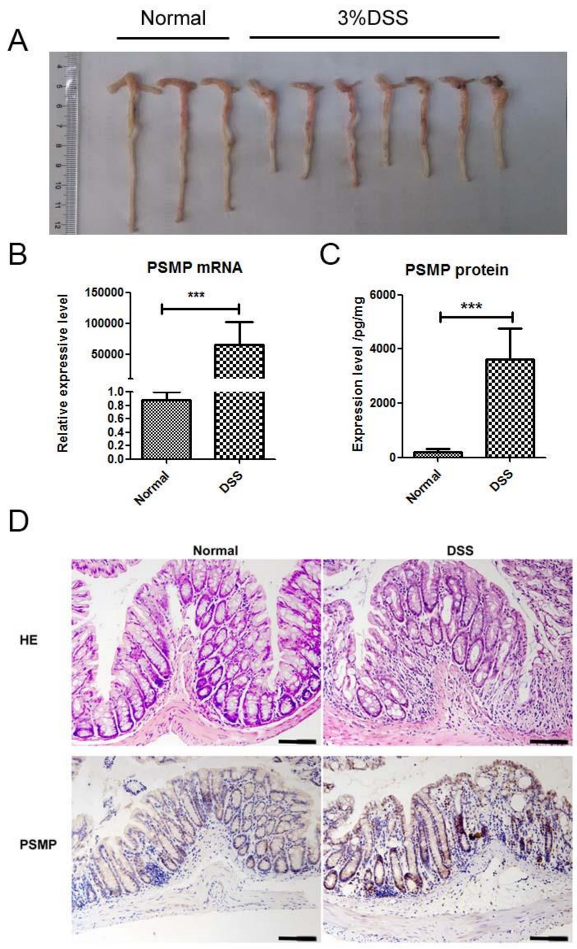

Figure S1. PSMP was up-regulated in the DSS-induced colitic tissue. (A) The mouse colons of the control (Normal) group and DSS-treated group. (**B) and (C)**The mRNA (B) and protein (C) levels of PSMP in the normal colons and DSS-induced colitic tissues were detected by real-time PCR or CBA and statistically analyzed. **(D)** The colonic tissue biopsies from the two groups were stained with HE or IHC with PSMP antibody; scale bars = 100 μm. *n* = 7 mice per group. *0.01<*p*<0.05, **0.001<*p*<0.01, and ****p*<0.001.

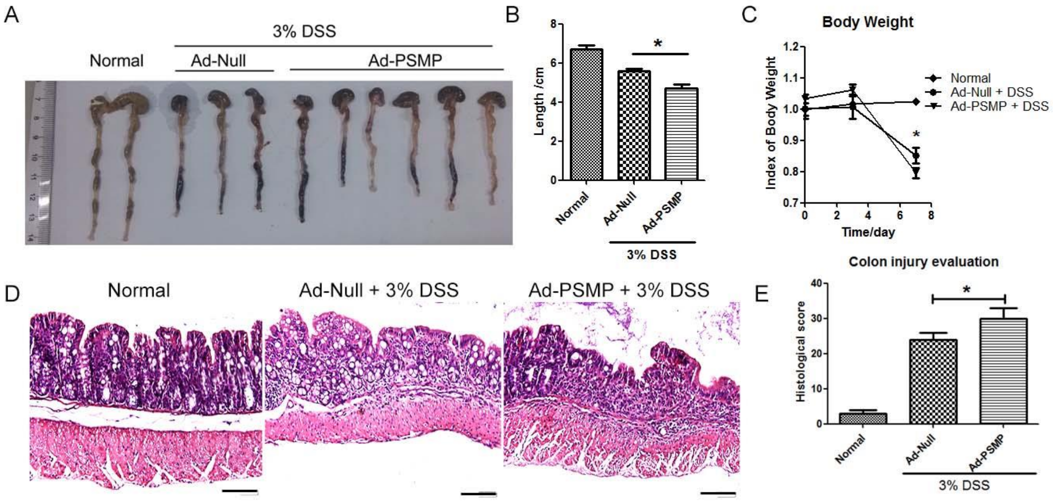

Figure S2. PSMP overexpression in the mouse colonic tissue aggravated DSS-induced colitis. (A) After the colons were infected with Ad-Null or Ad-PSMP *in situ* for 72 h, the normal group, the Ad-Null group and the Ad-PSMP group colons were separated. (**B) and (C)**The lengths of the colons and the body weight of the three groups were measured and statistically analyzed. (**D) and (E)** The colonic tissue biopsies from the three groups were stained with HE and then the histological score were assessed. Data were representative of at least two independent experiments with six mice per group. *0.01<p<0.05, **0.001<p<0.01, and ***p<0.001.

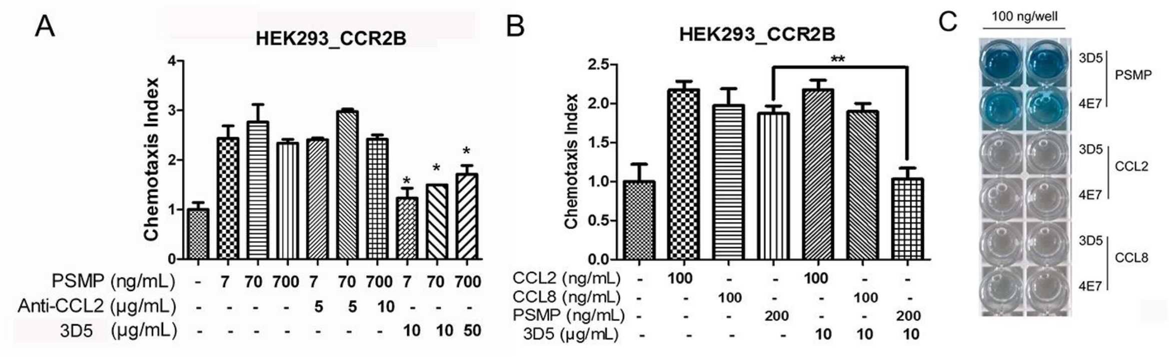

Figure S3. The monoclonal antibody 3D5 couldspecifically block and recognize PSMP. (A) and **(B)** Anti-PSMP neutralizing antibody 3D5 could block the chemotactic effect of PSMP, but not CCL2 and CCL8 in HER293 cells transfected with CCR2B. **(C)** Monoclonal antibody 3D5 could recognize PSMP, but not CCL2 and CCL8 by direct ELISA. Data were representative of at least three independent experiments.*0.01<*p*<0.05, **0.001<*p*<0.01, and ***p<0.001.

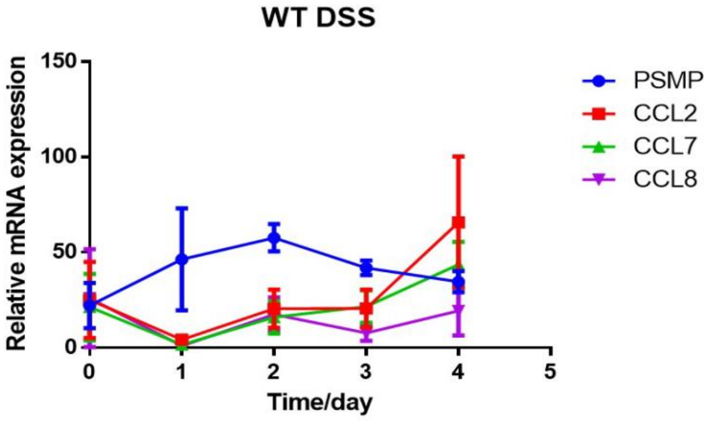

Figure S4. The time course of PSMP, CCL2, CCL7 and CCL8 expressions in DSS colitis tissue detected by real-time PCR. The primers of mouse Ccl2 were 5’-ATCCCAATGAGTAGGCTGGAGA’ (forward) and 5’-GGTGGTTGTGGAAAAGGTAGTGG -3’ (reverse). The primers of mouse Ccl7 were 5’-CCAATGCATCCACATGCTGC -3’ (forward) and 5’-TGCTATAGCCTCCTCGACCC -3’ (reverse). The primers of mouse Ccl8 were 5’-CCAATGCATCCACATGCTGC -3’ (forward) and 5’-TGCTATAGCCTCCTCGACCC -3’ (reverse). Data were representative of at least two independent experiments with four mice per group.*0.01<p<0.05, **0.001<p<0.01, and ***p<0.001.
